# Effects of Exercise on Blood Glucose and Glycemic Variability in Type 2 Diabetic Patients with Dawn Phenomenon

**DOI:** 10.1155/2020/6408724

**Published:** 2020-02-21

**Authors:** Xin Zheng, Yanyan Qi, Lina Bi, Wenli Shi, Yan Zhang, Dan Zhao, Su Hu, Meixin Li, Qin Li

**Affiliations:** ^1^Department of Endocrinology, China Rehabilitation Research Center, Beijing 100068, China; ^2^Department of Clinical Nutrition, China Rehabilitation Research Center, Beijing 100068, China; ^3^China Rehabilitation Science Institute, Beijing 100068, China; ^4^School of Rehabilitation Medicine, Capital Medical University, Beijing 100068, China

## Abstract

**Background:**

The dawn phenomenon (DP) is the primary cause of difficulty in blood glucose management in type 2 diabetic (T2D) patients, and the use of oral hypoglycemic agents has shown weak efficacy in controlling DP. Thus, this study is aimed at investigating the effect of moderate-intensity aerobic exercise before breakfast on the blood glucose level and glycemic variability in T2D patients with DP.

**Methods:**

A total of 20 T2D patients with DP confirmed via continuous glucose monitoring (CGM) participated in the current study. After collecting baseline measurements by CGM as a control, CGM was reinstalled and 30 minutes of moderate-intensity aerobic exercise was performed prior to breakfast. Dawn blood glucose increase, blood glucose levels, and glycemic variability were measured before and after exercise.

**Results:**

Dawn blood glucose increase (*Δ*Glu, 1.25 ± 0.84*vs.*2.15 ± 1.07, *P* = 0.005), highest blood glucose value before breakfast (Gmax, 8.01 ± 1.16*vs*. 8.78 ± 1.09, *P* = 0.005), highest blood glucose value before breakfast (Gmax, 8.01 ± 1.16*vs*. 8.78 ± 1.09, *P* = 0.005), highest blood glucose value before breakfast (Gmax, 8.01 ± 1.16*vs*. 8.78 ± 1.09, *P* = 0.005), highest blood glucose value before breakfast (Gmax, 8.01 ± 1.16*vs*. 8.78 ± 1.09, *P* = 0.005), highest blood glucose value before breakfast (Gmax, 8.01 ± 1.16*vs.*2.15 ± 1.07, *P* = 0.005), highest blood glucose value before breakfast (Gmax, 8.01 ± 1.16*vs.*2.15 ± 1.07, *P* = 0.005), highest blood glucose value before breakfast (Gmax, 8.01 ± 1.16*vs.*2.15 ± 1.07, *P* = 0.005), highest blood glucose value before breakfast (Gmax, 8.01 ± 1.16

**Conclusions:**

Acute moderate-intensity aerobic exercise before breakfast reduced the morning rise of blood glucose in T2D patients, partially counteracting DP. Furthermore, exercise significantly reduced blood glucose fluctuations and improved blood glucose control throughout the day. We recommend that T2D patients with DP take moderate-intensity aerobic exercise before breakfast to improve DP and glycemic control.

## 1. Introduction

The “dawn phenomenon” (DP) was first described by Schmidt in 1981 and refers to the spontaneous increase of blood glucose or insulin demand occurring at dawn, despite diabetic patients demonstrating reasonable blood glucose at night without hypoglycemia [1]. A patient is said to exhibit DP when the difference between the lowest blood glucose level at night and the highest blood glucose level before breakfast is ≥20 mg/dL (1.1 mmol/L) [2]. While elevated blood glucose from morning to breakfast is termed the “dawn phenomenon,” abnormal elevation of blood glucose after breakfast is termed “extended dawn phenomenon” [3]. DP and the extended DP are primary causes of difficulty in blood glucose management in diabetic patients, as the use of oral hypoglycemic agents alone, or in combination, have shown weak efficacy in controlling DP [3, 4].

Glycemic variability is an important indicator of blood glucose control. Specifically, glycemic variability refers to an unstable state in which glucose levels oscillate between peaks and troughs, including short-term blood glucose fluctuations (e.g., daytime glycemia and intraday blood glucose variations) and long-term blood glucose fluctuations (e.g., HbA1C variation) [5]. Glucose fluctuations increase the risk of diabetes complications [6, 7] by activating oxidative stress pathways that impair endothelial cell function and aggravate chronic inflammation and other vascular injuries [5, 8]. The risk associated with large swings in blood glucose is even greater than that of persistent hyperglycemia [9].

Continuous glucose monitoring (CGM) systems provide continuous, dynamic blood glucose testing, contributing to the discovery of hyperglycemia and hypoglycemia that are not easily detected by traditional monitoring methods [10]. CGM provides a possibility for the accurate diagnosis of the DP [11] and is the best means for evaluating glycemic variability [8]. As such, by examining the blood glucose profile provided by CGM, we can also understand the impact of exercise on blood glucose level and glycemic variability.

Exercise plays a crucial role in the integrated glycemia management of patients with type 2 diabetes (T2D). Regular exercise can increase insulin sensitivity, help blood glucose control, and prevent or delay the development of diabetes complications [12]. Guidelines recommend that adults with T2D should have at least 150 minutes of moderate-intensity aerobic exercise per week [13, 14].

Previous studies have primarily focused on the mechanism, influencing factors, and drug treatment of DP; however, it is not clear whether exercise can lead to improvement. In this study, CGM technology was applied to investigate blood glucose level changes and glycemic variability in T2D patients with DP after moderate-intensity aerobic exercise before breakfast, so as to provide new ideas for the intervention of DP in clinical practice.

## 2. Materials and Methods

### 2.1. Subjects

A total of 20 T2D patients with DP confirmed via CGM were recruited from the endocrinology department of the China Rehabilitation Research Center between August 2016 and September 2019. All research processes were performed at the center. Subjects were screened in two steps. First, multiple fingertip blood glucose monitoring was conducted for eligible subjects; this was performed at 3 AM along with fasting blood glucose (FBG). When the difference between FBG and nocturnal 3 AM blood glucose level was ≥1.1 mmol/L for three consecutive days, CGM was performed to further screen subjects with DP. All subjects met the diagnostic criteria for T2D defined by the WHO, while DP is defined as the difference between the lowest blood glucose level during the nocturnal period (Gmin) and the highest blood glucose value before breakfast (Gmax) of ≥20 mg/dL (1.1 mmol/L) [1, 2]. Exclusion criteria included type 1 diabetes, patients on insulin therapy, blood glucose < 3.9 mmol/L at night, uncontrolled hypertension, severe cardiopulmonary insufficiency, severe cerebrovascular disease, severe liver or kidney dysfunction, malignant neoplasms, thyroid disease, sleep disorders, sleep apnea, treatment with *β*-blockers, and individuals who were unable to complete aerobic exercise.

This study was approved by the Ethics Committee of China Rehabilitation Research Center; the institutional review board approval number is (2014)K(002). The study conformed to the ethical guidelines of the Declaration of Helsinki. All subjects provided written informed consent before participating.

### 2.2. Clinical Data Collection

Clinical information of the subjects was collected at the beginning of the study, including gender, age, height (m), body mass (kg), and body mass index (BMI, kg/m^2^). Blood pressure was obtained from the right upper arm with a mercurial sphygmomanometer after the subject rested for five minutes. After fasting overnight, each subject received venous blood tests measuring cholesterol (CHO), high-density lipoprotein (HDL), low-density lipoprotein (LDL), triglyceride (TG), and plasma glucose (FPG) by using an automatic analyzer (BS-2000M, Mindray, China). Fasting insulin (FINS) was measured by a chemiluminescence detector (I2000SR, Abbott, USA). Glycosylated hemoglobin (HbA1C) was measured with ion-exchange high performance liquid chromatography using an automatic glycohemoglobin analyzer (HLC-723G8, TOSOH, Japan). Insulin resistance levels were assessed via the HOMA-IR homeostasis model calculated by FPG (mmol/L)∗FINS (*μ*IU/L)/22.5. Beta cell function was assessed with HOMA-*β* and calculated by 20∗FINS (*μ*IU/L)/(FPG (mmol/L) − 3.5) (%) [15].

### 2.3. CGM Measures

Identical CGM monitors (CGMS GOLD, Medtronic MiniMed, USA) were used to monitor blood glucose. In order to avoid the deviation caused by the insertion and removal of the sensor, and to reduce the influence of daytime variability, the mean value of two complete 24-hour sessions of recorded data was calculated for subsequent comparison. Subjects were fitted with CGM monitors, and baseline CGM data were collected. After collecting baseline measurements by CGM as control, CGM was reinstalled and exercise was performed. After three days of exercise, the CGM was removed, and the mean value of the two complete 24 hour sessions of recorded data was analyzed again. Indicators, including dawn blood glucose increase, blood glucose levels, and glycemic variability, were measured before and after exercise. The rise of blood glucose at dawn (*Δ*Glu) equals Gmax − Gmin. The duration of the DP was defined as the time interval between the nadir and pre-breakfast time points. The blood glucose control level was evaluated by mean blood glucose (MBG), time in range (TIR), mean blood glucose 1 h before breakfast, mean blood glucose after breakfast (within 3 hours), and hyperglycemia area. Intraday glycemic variability was evaluated by blood glucose standard deviation (SD), coefficient of variation (CV), mean amplitude of glucose excursion (MAGE), the largest amplitude of glucose excursion (LAGE), low glycemic index (LBMI), and mean postprandial glucose excursion (MPPGE). Daytime glycemic variability was evaluated using daily differences (MODD).

### 2.4. Exercise Intervention

This was a self-controlled study before and after exercise intervention. After baseline CGM data were collected, all subjects were fitted with CGM monitors again and exercised before breakfast (06:27–07:00) on a treadmill (D847MLN, Technogym, Italy) with moderate intensity of 50–60% maximal oxygen uptake (VO_2max_) for three consecutive days. Target heart rate during exercise was equal to heart rate reserve multiplied by training intensity plus resting heart rate (heart rate reserve = maximal heart rate − resting heart rate and maximal heart rate = 220 − age). After 3 minutes of exercise, the target heart rate was reached, and subjects continued exercising for 30 minutes at the target heart rate. Additionally, warm-up and cool-down periods of five to ten minutes were performed before and after exercise, respectively. After the third day of exercise, we removed the CGM, analyzed the mean value of the two complete 24 hour sessions of recorded data, and then compared the results with the baseline indicators before exercise.

Three meals were provided by the clinical nutrition department at fixed times. The hypoglycemic regimen was unchanged during the experiment.

### 2.5. Statistical Analyses

All data were analyzed using SPSS 25.0 (SPSS Inc., Chicago, IL, USA). We selected two complete 24 hour sessions of recorded data provided by the CGM for each subject before and after exercise, and then analyzed the mean value of the two complete 24 hour sessions of recorded data to obtain the required indicators. Measurement data were expressed as the mean ± standard deviation (mean ± SD) or median and interquartile range (IQR), as appropriate. This was a self-controlled study before and after exercise. The paired *t*-test was used to compare Gmax, Gmin, *Δ*Glu, MBG, TIR, mean blood glucose before breakfast, mean blood glucose after breakfast, SD, CV, MAGE, LAGE, MPPGE, and MODD changes before and after exercise. Wilcoxon matched-pairs signed-rank tests were used to compare hyperglycemic area and LBMIs for non-normal distributions. A *P* value < 0.05 was considered statistically significant.

## 3. Results

### 3.1. Baseline Data

The baseline clinical and laboratory data of the subjects are shown in Table 1. Data indicated that BMI, HbA1C, FPG, and HOMA-IR levels of participants were higher than the normal range, and HOMA-*β* levels were lower than the normal range.

### 3.2. Blood Glucose Level and Glycemic Variability Index before and after Exercise

After exercise, values for Gmax (8.01 ± 1.16*vs*. 8.78 ± 1.09, *P* = 0.005), *Δ*Glu (1.25 ± 0.84*vs.*2.15 ± 1.07, *P* = 0.005), MBG (7.8 ± 0.97*vs*. 8.37 ± 0.95, *P* = 0.001), blood glucose 1 h before breakfast (7.5 ± 1.13*vs.*8.22 ± 1.06, *P* = 0.002), blood glucose after breakfast (8.41 ± 1.22*vs.*9.29 ± 1.21, *P* < 0.001), and hyperglycemia area (0 (0, 0.138) *vs.* 0.125 (0.013, 0.35), *P* = 0.006) were all lower and TIR (90.75 ± 12.27*vs*. 83.5 ± 15.41, *P* = 0.015) was higher than before exercise. By contrast, there was no difference observed in Gmin (Table 2).

Among the glycemic variability indicators, SD (1.1 ± 0.5*vs*. 1.48 ± 0.63, *P* = 0.001), CV (14.14 ± 5.94*vs.*17.69 ± 7.46, *P* = 0.006), MAGE (2.71 ± 1.52*vs.*3.73 ± 1.98, *P* = 0.006), LAGE (4.97 ± 2.07*vs.*6.41 ± 2.36, *P* = 0.002), MODD (1.02 ± 0.36*vs*. 1.23 ± 0.41, *P* = 0.03), and MPPGE (2.65 ± 1.55*vs*. 3.29 ± 1.79, *P* = 0.009) were all lower after exercise. However, there was no difference observed in LBMI before and after exercise (Table 3).

According to the criteria recommended by the Chinese Clinical Practice Guideline for continuous glucose monitoring, in which the normal values are MBG < 6.6, SD < 1.4, and MAGE < 3.9 [16], the MBG of all 20 patients did not meet the standard, while the SD and MAGE of 13 patients were up to the standard. After exercise, the MBG of three additional patients, the SD of three additional patients, and the MAGE of four additional patients reached the standard. Furthermore, the DP of 10 patients disappeared completely.

## 4. Discussion

The pathophysiological mechanism of the DP has not yet been fully established. At dawn, the secretion of insulin antagonistic hormones, such as growth hormone, cortisol, and glucagon, increase. Current research suggests that the DP is the result of the combination of increased endogenous glucose production and persistent insulin resistance in the early morning due to islet *β*-cell dysfunction [17, 18]. Abnormal hyperglycemia caused by the DP is not only limited to fasting, but also largely affects blood glucose levels after breakfast, i.e., the extended DP. Monnier et al. found that blood glucose levels before breakfast in patients with T2D without insulin treatment increased by an average of 0.89 mmol/L compared with the lowest value of nocturnal blood glucose, and blood glucose levels after breakfast are higher than that after lunch and dinner [2]. The effects of the DP and extended DP on total daily blood glucose and HbA1C were 0.67 mmol/L and 0.4%, respectively, which were not affected by the treatment regimens [2, 4]. DP, one of the causes of elevated FBG, can cause an increase in blood glucose levels throughout the day, resulting in poor overall glycemic control [11, 19]. The primary goal of treatment of DP is to reconstruct near-normal blood glucose values before and after breakfast to reduce daily mean blood glucose and HbA1C levels [2, 4].

In this study, we observed that moderate aerobic exercise before breakfast helped to reduce the rise of blood glucose at dawn that often occurs in type 2 diabetes patients. In particular, the significant improvement of blood glucose one hour before breakfast is strong evidence that exercise before breakfast can improve fasting hyperglycemia. Blood glucose was also significantly improved after breakfast, suggesting that exercise before breakfast further improved instances of extended DP. TIR is defined as the proportion of time in which blood glucose is in the range of 3.9 to 10.0 mmol/L during a 24-hour period. CGM technology can accurately obtain TIR. In the International Consensus on CGM Applications in 2017, TIR was recommended as one of the indicators for CGM standardization reports [20]. Studies have shown that TIR is significantly associated with the risk of retinopathy in patients with T2D and is independent of HbA1C, suggesting that TIR can be a new indicator to evaluate blood glucose control [21]. This study also observed that TIR and MBG significantly improved after exercise, and that the area of hyperglycemia decreased, suggesting that exercise before breakfast improved the control of blood glucose throughout the day.

The acute effects of exercise are beneficial for patients with T2D. Exercise increases the concentration of GLUT-4 in the cell membrane and increases glucose uptake in skeletal muscles [22–24]. Acute exercise increases glucose tolerance and insulin sensitivity and blood glucose reduction within 20–72 h, depending on the exercise intensity, form, duration, and observation time [25–33]. Studies have shown that acute moderate-intensity endurance training leads to a reduction in blood glucose levels [25, 30], whereas light and short resistance and endurance training shows no effect [30, 31]. Aerobic exercise is more effective than resistance exercise in acute hypoglycemic effects [32, 33]. This study involved three consecutive days of moderate-intensity aerobic exercise, which was confirmed to be the most effective exercise program.

Many studies have shown that fluctuating hyperglycemia more severely damages *β* cells than persistent hyperglycemia [5, 7, 8]. Acute blood glucose fluctuations can also accelerate diabetic macrovascular and microvascular diseases by exacerbating oxidative stress and interfering with intracellular and extracellular signaling pathways [5, 7, 34]. Glycemic variability also increases the risk of hypoglycemia and increases mortality [7, 8]. As such, more attention is being paid to the assessment of glycemic variability in diabetic patients. SD is the difference between blood glucose level and the mean value; the higher the value, the greater the fluctuation of blood glucose. CV is the ratio of SD to MBG, which can eliminate the influence of different mean levels on the degree of variation. MAGE is considered the “gold standard” for assessing glycemic variability and is widely used in clinical research. MAGE is characterized by filtering small fluctuations whose amplitude does not exceed a certain threshold, thus reflecting the variation of blood glucose rather than discrete features, which does not depend on the overall level of blood glucose. LAGE is the difference between the maximum and minimum intraday blood glucose values, reflecting the maximum blood glucose fluctuation during the day. MPPGE reflects postprandial blood glucose stability. MODD is the mean of the absolute difference between two consecutive 24 hour glycemic spectra that are matched with blood glucose, resulting in a reflection of daytime blood glucose fluctuations [8, 35]. Although exercise plays an important role in the treatment of T2D, the effects of exercise on glucose variability remain unclear. One study showed that acute aerobic exercise and combined exercise can reduce glucose variability in patients with T2D [25]. This study observed that SD, CV, MAGE, LAGE, MODD, and MPPGE were significantly reduced after moderate-intensity aerobic exercise before breakfast, indicating that moderate-intensity aerobic exercise before breakfast helps to lower blood glucose fluctuations. This result is of great significance for the overall stable control of blood glucose, which may further delay the occurrence and development of diabetic complications.

The timing of exercise has also become a controversial issue in recent years [36]. The 2016 American Diabetes Association (ADA) Position Statement: Physical Activity/Exercise and Diabetes and the 2019 ADA Diabetes Diagnosis and Treatment Standard Recommendations on Physical Activity have no clear requirements for pre-meal or post-meal exercise [12, 14]. Studies have shown that in healthy subjects, morning exercise is more effective in the fasting state than the same amount of exercise in the fed state [37]. Fasting exercise reduces the accumulation of harmful fatty acids by accelerating lipid breakdown of muscle cells, which helps to improve peripheral insulin sensitivity and better improves glucose tolerance [37]. Acute endurance training with fasting in healthy subjects is accompanied by lower blood insulin, higher blood free fatty acid concentrations, stable blood glucose levels (at least within 60–90 minutes before exercise), accelerated lipid oxidation in muscle cells, systemic fat decomposition, and muscle glycogen storage, while long-term endurance training in a fasting state is accompanied by more considerable improvement in insulin sensitivity and muscle fat oxidation capacity [38]. Endurance training in a fasting state in patients with T2D requires further study. In this study, there was no statistical difference in LBMI before and after exercise. The LBMI value refers to a comprehensive analysis of the frequency and degree of hypoglycemia and is an accurate assessment of the risk of severe hypoglycemia through the corresponding mathematical treatment of blood glucose measurements [35]. No subjects exhibited hypoglycemia before, after, or during exercise, which verified the safety and effectiveness of exercise before breakfast for such patients with DP. At the same time, this study embodies the principle of individualization necessary when prescribing exercise to patients.

## 5. Conclusions

In summary, acute moderate-intensity aerobic exercise before breakfast appeared to counteract DP, significantly reduced blood glucose fluctuations, and improved blood glucose control throughout the day. To the best of our knowledge, our research provides the first evidence showing this potential by exercise. This study provides a new concept for T2D patients to ameliorate DP, reduce blood glucose fluctuation, and achieve overall stable blood glucose control. However, whether the observed improvement was due to the simple hypoglycemic effect of exercise or the additional improvement effect of exercise on the mechanism of the DP is not yet clear. Thus, it is necessary to explore the mechanism of improving the DP by further research that considers increasing the number of cases, exercising for a longer duration, and increasing observations of relevant hormone indices.

## Figures and Tables

**Table 1:** Baseline characteristics of all subjects.

Patients tested (*n*)	20
Sex (M/F)	11/9
Age (y)	50.75 ± 9.36
Diabetes duration (y)	5.88 ± 4.03
SBP (mmHg)	129.75 ± 11.17
DBP (mmHg)	82.60 ± 8.49
BMI (kg/m^2^)	25.86 ± 3.85
TC (mmol/L)	4.56 ± 1.10
HDL-C (mmol/L)	1.05 ± 0.18
LDL-C (mmol/L)	2.81 ± 0.90
TG (mmol/L)	1.90 ± 0.90
HbA1C (%)	9.10 ± 1.80
FPG (mmol/L)	9.39 ± 2.38
FINS (*μ*IU/mL)	7.54 ± 4.24
HOMA-IR	3.13 ± 1.78
HOMA-*β* (%)	19.11 (16.84, 37.77)

**Table 2:** Comparison of blood glucose level before and after exercise.

	Gmin (mmol/L)	Gmax (mmol/L)	*Δ*Glu (mmol/L)	Duration of DP (h)	MBG (mmol/L)	TIR (%)	Before breakfast (mmol/L)	After breakfast (mmol/L)	Hyperglycemic area (mmol/L∗day)
Before	6.63 ± 1.08	8.78 ± 1.09	2.15 ± 1.07	4.84 ± 1.57	8.37 ± 0.95	83.5 ± 15.41	8.22 ± 1.06	9.29 ± 1.21	0.125(0.013,0.35)
After	6.79 ± 1.22	8.01 ± 1.16	1.25 ± 0.84	4.40 ± 1.65	7.8 ± 0.97	90.75 ± 12.27	7.5 ± 1.13	8.41 ± 1.22	0 (0, 0.138)
*t* (*z*)	-0.627	3.149	3.193	1.163	3.841	−2.662	3.677	4.272	-2.769
*P*	0.538	0.005	0.005	0.259	0.001	0.015	0.002	<0.001	0.006

Gmin: lowest blood glucose level during the nocturnal period; Gmax: highest blood glucose value before breakfast; duration of DP: the time interval between the nadir and prebreakfast time points; ΔGlu = Gmax–Gmin; MBG: mean blood glucose; TIR: time in range.

**Table 3:** Comparison of glycemic variability indexes before and after exercise.

	SD	CV (%)	MAGE	LAGE	LBMI	MODD	MPPGE
Before	1.48 ± 0.63	17.69 ± 7.46	3.73 ± 1.98	6.41 ± 2.36	0.17 (0, 0.49)	1.23 ± 0.41	3.29 ± 1.79
After	1.1 ± 0.5	14.14 ± 5.94	2.71 ± 1.52	4.97 ± 2.07	0.2 (0, 0.37)	1.02 ± 0.36	2.65 ± 1.55
*t* (*z*)	4.09	3.069	3.07	3.598	-0.414	2.347	2.895
*P*	0.001	0.006	0.006	0.002	0.679	0.03	0.009

SD: blood glucose standard deviation; CV: blood glucose coefficient of variation; MAGE: mean amplitude of glucose excursion; LAGE: largest amplitude of glucose excursion; LBMI: low glycemic index; MPPGE: mean postprandial glucose excursion; MODD: mean of daily differences.

## Data Availability

The data used to support the findings of this study are available from the corresponding author upon request.
